# The role of ferroptosis in prostate cancer: a novel therapeutic strategy

**DOI:** 10.1038/s41391-022-00583-w

**Published:** 2022-09-02

**Authors:** Yue Wang, Yifan Ma, Kui Jiang

**Affiliations:** 1 Department of Medical Oncology, The Second Affiliated Hospital of Dalian Medical University, Dalian, Liaoning China; 2 Department of Neurology, The Second Affiliated Hospital of Dalian Medical University, Dalian, Liaoning China

**Keywords:** Diseases, Cancer

## Abstract

The incidence of prostate cancer is the second most among male cancers after lung cancer. Prostate cancer develops rapidly and is inclined to metastasize, and castration-resistant prostate cancer (CRPC) can be formed in the later stage, which brings great challenges to the prognosis and treatment. At present, the main treatment of prostate cancer is generally divided into four methods: surgery, chemotherapy, radiotherapy and endocrine therapy. However, the efficacy of these methods fails to satisfy the demands of patient prognosis. Ferroptosis is a newly discovered iron-dependent process, characterized by lipid peroxidation. Ferroptosis is associated with many diseases, especially tumor growth. In recent years, inhibiting tumor growth and overcoming tumor drug resistance by inducing ferroptosis has become a hot research topic. Previous studies have shown that induction of ferroptosis may be a new treatment for prostate cancer. We review the research progress of ferroptosis in prostate cancer in order to provide highly effective therapies for patients with prostate cancer.

## Introduction

Prostate cancer is the most common type of cancer among male urogenital system tumors [1]. Prostate cancer is characterized by abnormal cell division in the prostate, resulting in abnormal growth of the prostate. Most men don’t die from prostate cancer, but are affected by slow-growing tumors. The death of prostate cancer is mainly due to the spread of cancer cells to other parts of the body, such as bone, pelvic, lumbar vertebra, bladder, rectum, and brain [2, 3]. Currently, there are only three recognized independent risk factors for prostate cancer: age (i.e., increased risk of prostate cancer with age), race (i.e., increased risk of African Americans) and genetic factors (hereditary factor) [4]. The incidence rate of prostate cancer is positively correlated with age. As life expectancy increases, so does the incidence of prostate cancer. According to different types of prostate cancer, there are four key treatment methods: radical prostatectomy, chemotherapy, androgen deprivation and radiotherapy [5]. These can improve the early survival rate of patients with prostate cancer. However, most patients were found in the late stage with metastasis, which made the prognosis worse. Androgen deprivation therapy (ADT) has always been the basic treatment for advanced and metastatic prostate cancer. Despite the high initial response rate, most of advanced patients eventually develop progressive prostate cancer after ADT. This is called castration-resistant prostate cancer (CRPC) [6]. At the same time, the drastic reduction of serum testosterone levels induced by ADT produces multiple side effects such as bone fracture, cardiovascular disease, sexual dysfunction anemia. It reduces the quality of patients’ life [7–10]. Therefore, it is particularly important to develop new methods for treat prostate cancer. In 2012, Dixon et al. found that the antitumor drug erastin induces unique iron dependent non-apoptotic cell death in Ras mutant tumors. This cell death process cannot be inhibited by regulatory cell death specific inhibitors, but antioxidants and chelators can prevent and reverse this process. They proposed the term ‘ferroptosis’ to describe a new mode of regulated death distinct from apoptosis, autophagy and necrosis, characterized by lipid peroxidation dependent on iron [11]. The excessive accumulation of lipid reactive oxygen species (ROS) leads to destroying the cell membrane, leading to cell death [12]. It was observed that mitochondria are smaller than normal, membrane density increased and mitochondrial cristae decreased [11].

Ferroptosis is considered to be associated with a variety of human diseases, such as neurodegeneration, ischemia-reperfusion injury, and various cancers, including prostate cancer [13–17]. Tumor cells are more dependent on iron than normal cells for their high proliferation rate. This phenomenon is called iron addiction [18]. The discovery of ferroptosis makes people have a new understanding of the occurrence and development of tumor diseases. There is increasing evidence that ferroptosis leads to tumor growth inhibition. Using inducers to induce ferroptosis or regulate ferroptosis-related genes may become an anti-cancer strategy. Therefore, it is great significance to understand the mechanism of ferroptosis and its research progress in prostate cancer.

## The main mechanism of ferroptosis

### Inhibiting the cysteine-glutamate transporter system Xc^−^ can induce ferroptosis

System Xc^−^ is a sodium independent antiporter that export intracellular glutamate and import extracellular cystine across the membrane in a ratio of 1:1 to synthesize glutathione in cells [19]. It consists of two subunits, SLC7A11 and SLC3A2. SLC7A11 is connected to SLC3A2 through a disulfide bond between the conserved residue cys158 of SLC7A11 and cys109 of SLC3A2 [20]. A recent study showed that the CD44 variant subtype (CD44v) also interacts with and stabilizes SLC7A11 on the surface of cancer cells [21]. SLC7A11 is a multichannel transmembrane protein that serves as a functional component of system Xc^−^. SLC3A2, a single transmembrane protein, is the molecular chaperone that maintains SLC7A11 protein stability and proper membrane localization [20]. Novel small molecules such as erastin and sorafenib have been identified as system Xc^−^ inhibitors to promote ferroptosis [22, 23]. Cadmium (Cd) is a toxic metal element and a pollutant existing in the environment. Cd exposure is primarily through the intake of contaminated food and water, and largely through inhalation and smoking. International cancer and other epidemiological research institutions suggest that Cd can lead to prostate cancer [24]. Zhang et al. found that chronic cadmium exposure inhibited ferroptosis and promoted the proliferation of prostate cancer cell. RNA sequencing revealed that lncRNA OIP5-AS1 was significantly up-regulated in the proliferation of prostate cancer induced by Cd exposure. OIP5-AS1 inhibits ferroptosis by miR-128-3p/SLC7A11 axis [25]. As a tumor suppressor gene, p53 plays an important role in inhibiting tumor growth [26]. Recent study have found that p53 is involved in ferroptosis, which inhibits cystine uptake by inhibiting the expression of SLC7A11 and sensitizing cells to ferroptosis [27]. Flubendazole inhibits the proliferation of CRPC by inhibiting the expression of SLC7A11 by inducing p53, further downregulating glutathione peroxidase 4 (GPX4), and promoting ferroptosis. In addition, flubendazole displayed a synergistic effect with 5-fluorouracil (5-FU) in CRPC chemotherapy. After combined use, it further promotes the decrease of SLC7A11 expression to promote ferroptosis and enhance the drug effect [28].

### Inhibiting the activity of GPX4 can induce ferroptosis

GPX4 is an antioxidant enzyme that protects cells and membranes from peroxidation by using glutathione as a cofactor to protect cells from lipid peroxidation. Glutathione can cycle between reduced (GSH) and oxidized (GSSG) states, enabling this metabolite to participate in redox biochemical reactions [29, 30]. ChaC glutathione specific γ-glutamylcyclotransferase 1 (CHAC1) can decrease the content of intracellular GSH to prompt ferroptosis in prostate cancer cell with increasing the sensitivity of prostate cancer cells to docetaxel [31]. Inhibition of GPX4 can lead to accumulation of ROS, accompanied by lipid peroxidation, and eventually lead to ferroptosis [32]. Besides, GPX4 can reduce toxic lipid peroxides (e.g., R–OOH) to correspond lipid alcohols (e.g., R–OH). RSL3 can covalently inactivate GPX4 by binding to selenocysteine in the active site of GPX4 [33]. GPX4 is the core inhibitor of ferroptosis. Recent studies have reported that serum miRNA is a promising target for cancer research and therapy. MiRNAs mainly induce the degradation of mRNAs or inhibit their translation by interacting with the ‘3’-UTR of target mRNAs, leading to changes in regulatory factors in cellular physiological processes. Down regulation of miR-15a expression was observed in patients with prostate cancer. MiR-15a can interact with the 3 ‘- untranslated region (UTR) of GPX4 mRNA to negatively regulate the expression of GPX4. The use of miR-15a mimic or siGPX4 can promote the death of prostate cancer cells [34]. SLC7A11 and GPX4 are highly expressed in advanced prostate cancer cells. The use of ferroptosis activator erastin or RSL3 can promote cancer cell death by inducing ferroptosis. The second-generation anti-androgen drugs such as enzalutamide or abiraterone in standard treatment of advanced prostate cancer combined with ferroptosis activator can further inhibit tumor proliferation [17].

### ROS production is essential for ferroptosis

ROS are usually composed of superoxide, peroxides and free radicals [35]. As unstable molecules, they are produced in living cells as normal metabolites which play a significant role in signal transduction and maintaining tissue homeostasis [36]. ROS are involved in various physiological or pathological processes, such as metabolism, inflammation, neurogeneration and carcinogenesis [37–40]. When cells respond to oxidative stress, large amounts of highly reactive and toxic ROS are produced and lead to adverse changes in cellular components such as proteins, lipids, and DNA damage [41].Cell membrane is particularly vulnerable to ROS damage due to its high polyunsaturated fatty acids (PUFA), called “lipid peroxidation”, which is the most significant feature of ferroptosis. Compared with normal cells, cancer cells are more vulnerable to ferroptosis and ROS accumulation. Cisplatin is a widely used anticancer drug. The resistance to cisplatin is an important obstacle to chemotherapy in patients with prostate cancer. The ferroptosis activator RSL3 increases the sensitivity of prostate cancer cells to cisplatin by producing ROS, aggravating cell cycle arrest and apoptosis caused by cisplatin [42]. Diallyl trisulfide (DAT) is kind of the main decomposition products and active components of allicin. Studies have found that DAT has a variety of biological effects, such as anti-tumor, bacteriostasis, antioxidant stress and participation in the regulation of inflammatory response. In prostate cancer, it causes an increase in reactive oxygen species, accompanied by ferritin degradation to prompt ferroptosis, and inhibits the growth of cancer cells [43]. Artemisinin was first extracted from Artemisia annua in 1971 by Tu youyou [44]. It is a semiterpene lactone with antimalarial effect. In recent years, it has been found that it has anticancer effect and plays an important role in inducing ferroptosis. Artemisinin can induce ferroptosis in prostate cancer cell DU145, but no similar effect was observed in PC3 and LNCaP cell lines [45]. Dihydroartemisinin (DHA) is an active metabolite of artemisinin. Numerous of studies have shown that DHA is cytotoxic to a variety of cancer cells, such as lung cancer, glioma cancer and so on [46, 47]. It can induce cancer cell ferroptosis, autophagy, and inhibit the proliferation of cancer cells. At present, there is no research to prove its role in prostate cancer ferroptosis, which can be a future research direction. Traditional Chinese medicine usually contains a variety of active components, which can produce additive or synergistic effects at the same time. Compared with targeted drugs, traditional Chinese medicine has the characteristics of multiple targets and can regulate a variety of signal pathways, such as regulating ADAMTS18, ROS, Nrf2, GPX4 and other molecules, so as to regulate ferroptosis. Using traditional Chinese medicine to induce ferroptosis in prostate cancer cells may become a future research direction.

### Iron-mediated oxidative damage in ferroptosis

Iron as a cofactor is important for maintaining a range of biological processes [48]. Iron overload can lead to fatal ROS production and lipid peroxidation [49]. Transferrin receptor 1(TFR1) is a transmembrane glycoprotein responsible for importing iron which is stored and transported in the form of the iron–protein complex (mainly ferritin) [50]. Iron oxide reductase steam3 (STEAP3) reduces iron in the form of Fe^3+^ to iron in the form of Fe^2+^ [18]. Finally, Fe^2+^ is released from divalent metal transporter 1 (DMT1) mediated endosome into unstable iron pool in the cytoplasm [51]. As an important factor in the formation of ROS through enzymatic or non-enzymatic reactions, iron plays an essential role in sensitizing cells to ferroptosis. Bordini et al. found that high dose of iron can inhibit the proliferation of prostate cancer cells through oxidative damage. In bicalutamide resistant cells, iron showed synergistic effect with bicalutamide [52]. In recent years, many studies have explored the mechanism of ferroptosis induced by ferroptosis inducers. The possible signal pathways and targets are shown in Table 1.

**Table 1:** Summary of ferroptosis inducers.

Ferroptosis inducers	Targets	Mechanisms	References
Erastin	SLC7A11	Inhibit System xc^−^ and prevent cystine import	[22, 23]
RSL3	GPX4	Inhibit GPX4 covalently	[33]
FIN56	GPX4	Autophagy-mediated GPX4 degradation	[63]
Sorafenib	SLC7A11	Inhibit System xc^−^ and prevent cystine import	[22, 23, 64]
Artesunate	FTH1,NCOA4	Promote degradation of FTH1 and NCOA4, elevate Fe^2+^ level, decrease GSH content	[65]
Dihydroartemisinin	GPX4, Mtor	Inhibit GPX4 directly, activate autophagy to degrade ferritin, elevate Fe^2+^ level	[66, 67]
Sulfasalazine	SLC7A11	Inhibit System xc^−^ and prevent cystine import	[68]
Diallyl trisulfide	GPX4	Promote production of ROS, degradation of ferritin protein, increase the labile iron pool	[43]
Cisplatin	GPX4	GSH depletion, lipid peroxidation	[69]
Curcumin	HO-1	Accumulation of intracellular iron, increase ROS level, decrease GSH content	[70]
Flubendazole	P53	P53 binds to SLC7A11 promoter region, inhibit System xc^−^	[28]

## Ferroptosis is a new direction for prostate cancer treatment

The phosphatase and tensin homolog (PTEN) deleted on chromosome 10 gene is a tumor suppressor gene discovered in recent years. Its product PTEN protein has lipid phosphatase activity and protein phosphatase activity. PTEN exerts its anti-tumor effect mainly by acting on the downstream target molecule PIP3 of PI3K through its lipid phosphatase activity, thereby blocking the PI3K/Akt signaling pathway [53]. Sterol regulatory element-binding protein 1 (SREBP1) is a key transcription factor regulating lipid metabolism and encodes multiple genes for key enzymes in the adipogenesis pathway (such as SCD, FASN and ACLY). It was found that PI3K activation or PTEN gene deficiency promotes SREBP1/SCD mediated adipogenesis by activating PI3K/AKT/mTOR pathway to suppress ferroptosis. Inhibiting mTOR may be a new method for the treatment of prostate cancer [54].

The ferroptosis-related genes AIFM2 and NFSI were identified in a prostate cancer gene risk model, and in vivo and in vitro experiments showed that knockout of these two genes promotes ferroptosis [55]. Besides, pannexin2 (PANX2) is high expression in prostate cancer. Knocking out this gene promotes ferroptosis and inhibits prostate cancer cell growth [56]. Intriguingly, the discovery of database mining shows that ferroptosis-related genes are promising prognostic biomarkers and potential drug targets in prostate cancer patients. The growth of prostate cancer cells depends on the continuous activation of androgens, the androgen receptor (AR) and its splice variants, which remain the main driver of CRPC progression. As a classical inducer of ferroptosis, erastin can inhibit the transcriptional activity of AR and its splice variants in vitro and in vivo. In addition, when erastin was combined with docetaxel in the treatment of CRPC, the growth inhibitory effect of docetaxel was found to be enhanced. In vivo experiments, erastin can further enhance the antitumor effect of docetaxel, and there is no obvious damage to various organs of mice, with less toxic and side effects, which can provide experimental basis for clinical research [57]. Li et al. also found that anti androgen combined with ferroptosis activator RSL3 inhibited the growth of prostate cancer cells in mouse xenografts [17]. Further clinical trials can be conducted in the future to prove the role of ferroptosis in the treatment of prostate cancer. Meanwhile, 2,4-Dienoyl-CoA reductase (DECR1) was discovered when analyzing genes associated with AR inhibitor resistance. DECR1 is a target gene negatively regulated by AR. When this gene is knocked out, it promotes ferroptosis in CRPC cells [58]. In the latest study, isothiocyanate (ITC)-containing AR antagonists were synthesized, which downregulates AR and its spliceosome. Combination with BSO, a GSH inhibitor promotes lipid peroxidation and ferroptosis in prostate cancer cells [59]. Kumar et al. showed that supraphysiological testosterone can inhibit tumor proliferation by producing lipid peroxides, targeting prostate cancer cell associated lipid metabolism to inhibit prostate cancer cell growth is considered as a possible therapeutic strategy [60].

It is worth mentioning that recent studies have found that endoplasmic reticulum stress response plays an important role in ferroptosis. On the one hand, the activation of endoplasmic reticulum stress pathway in cancer cells can inhibit ferroptosis and participate in the induction of drug resistance. On the other hand, endoplasmic reticulum stress can promote cell ferroptosis and may be involved in the co-regulation of ferroptosis and apoptosis [61]. Some studies have also shown that ferroptosis inducers can activate ERK-eIF2 mediated by endoplasmic reticulum stress ATFα-ATF4-CHOP cascade without inducing apoptosis. LNCaP-AI cells have higher ATF6 expression than LNCap-A cells. The highly expressed ATF6 mediates tolerance to ferroptosis through transcriptional activation of PLA2G4A, and inhibition of ATF6α signaling by Ceapin-A7 enhances the effect of enzalutamide on CRPC xenograft growth [62]. Understanding the relationship between ferroptosis and endoplasmic reticulum stress, apoptosis, autophagy is of great significance for overcoming the drug resistance of cancer cells. However, little research has been done in this area of prostate cancer. Whether such mutual regulation exists in prostate cancer remains to be further explored.

## Discussion

**Fig. 1:**
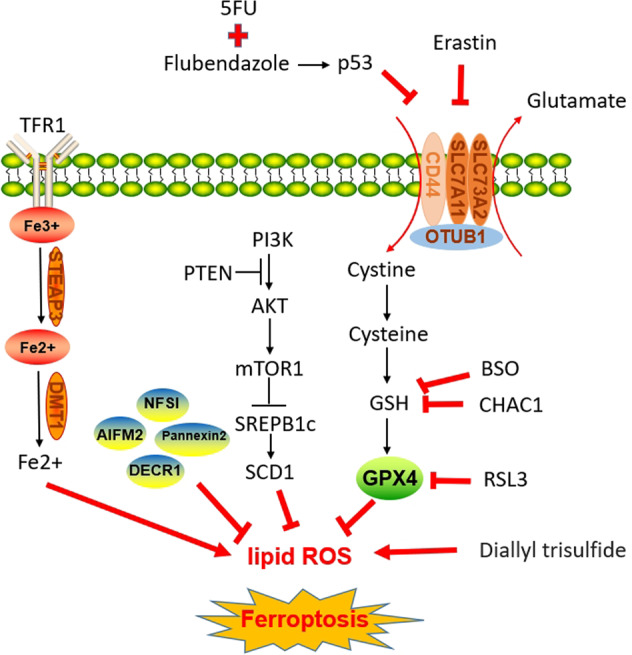
The main mechanism of ferroptosis in prostate cancer. The system Xc^−^ exports intracellular glutamate and transmembrane import of extracellular cystine, which is then converted to cysteine for GSH synthesis. Erastin and Flubendazole can inhibit the activity of System Xc^−^. GPX4 can inhibit the accumulation of ROS with the help of GSH. CHAC1 and the GSH inhibitor BSO can inhibit intracellular GSH to prompt ferroptosis. RSL3 can inhibit the activity of GPX4. Circulating iron is bound to transferrin in the form of Fe3+ and then enters cell via TFR1. STEAP3 reduces iron in the form of Fe3+ to iron in the form of Fe2+. Finally, Fe2+ is released from DMT1 to the unstable iron pool in the cytoplasm to mediate lipid ROS. Knockdown of AIFM2, Pannexin2, DECR1 and NFSI can improve lipid ROS. Carcinogenic activation of PI3K/AKT/mTOR1 signaling suppresses ferroptosis through SREBP1/SCD1-mediated lipogenesis.

In 2012, Dixon et al. proposed the term ferroptosis to describe an iron-dependent regulatory form of cell death caused by accumulation of lipid reactive oxygen species [11]. We review the important role of ferroptosis in prostate cancer. Multiple drugs combined with ferroptosis inducers can enhance their anticancer effects. For example, flubendazole combined with 5-FU can induce CRPC death by promoting ferroptosis [28]. The second-generation antiandrogens combined with ferroptosis activators can further inhibit tumor proliferation by inhibiting the expression of GPX4 [17]. Ferroptosis activators can also make prostate cancer cells more sensitive to DDP [42]. In the latest study, isothiocyanate (ITC)-containing AR antagonists were synthesized which can down regulates AR and its spliceosomes. When combined with BSO, it can promote ferroptosis in prostate cancer cells [58]. Although these studies revealed some important findings, we have already explored only parts of the mechanism of ferroptosis in prostate cancer (Fig. 1), which has not been extensively studied in prostate cancer. Whether there are other important mechanisms of ferroptosis remains unclear. Through database analysis, many ferroptosis-related genes may be associated with prostate cancer, but the specific mechanisms by which these genes affect prostate cancer cells are unclear. Targeting these genes to induce ferroptosis may be a new therapy for prostate cancer. Several in vivo experiments have proved that the combination of ferroptosis activators can inhibit the proliferation of prostate cancer cells [17, 57]. Further clinical experiments can be carried out in the future to prove the role of ferroptosis in the treatment of prostate cancer. Different prostate cancer cell lines show different sensitivities to ferroptosis. Therefore, we should consider this factor and how to address cellular tolerance to ferroptosis before using ferroptosis-inducing agents to treat prostate cancer. Whether there are more FDA-approved drugs that can induce ferroptosis in prostate cancer for clinical use or whether other unapproved drugs can induce ferroptosis in the treatment of prostate cancer will be the direction of our future exploration.

## Data Availability

The datasets used in this review are available from the corresponding authors on reasonable requests.
